# Three-dimensional tracking and analysis of ion channel signals across dendritic arbors

**DOI:** 10.3389/fncir.2013.00061

**Published:** 2013-04-04

**Authors:** Melanie Ginger, Philip Broser, Andreas Frick

**Affiliations:** ^1^NeuroCentre Magendie, Physiopathologie de la Plasticité Neuronale, INSERM U862Bordeaux, France; ^2^Physiopathologie de la Plasticité Neuronale, University of Bordeaux, INSERM U862Bordeaux, France; ^3^Department of Paediatric Neurology and Developmental Medicine, University of Tübingen Children's HospitalTübingen, Germany

**Keywords:** dendrites, ion channels, reconstruction, morphology, confocal microscopy, quantitative approaches, fluorescence intensity profiles

## Abstract

Most neuron types possess elaborate dendritic arbors that receive and integrate excitatory and inhibitory inputs from numerous other neurons to give rise to cell-type specific firing patterns. The computational properties of these dendrites are therefore crucial for neuronal information processing, and are strongly determined by the expression of many types of voltage-gated ion channels in their membrane. The dendritic distribution patterns of these ion channels are characteristic for each ion channel type, are dependent on the neuronal identity, and can be modified in a plastic or pathophysiological manner. We present a method that enables us to semi-automatically map and quantify in 3D the expression levels of specific ion channel types across the entire dendritic arbor. To achieve this, standard immunohistochemistry was combined with reconstruction and quantification procedures for the localization and relative distribution of ion channels with respect to dendritic morphology. This method can, in principle, be applied to any fluorescent signal, including fluorescently tagged membrane proteins, RNAs, or intracellular signaling molecules.

## Introduction

In most central nervous system (CNS) neurons synaptic inputs are widely distributed across extensive dendritic arbors. The role of the dendrites is to integrate this information to give rise to cell-type specific action potential patterns. Over the last 20 years or so it has become clear that the integrative properties of dendrites are strongly determined by the presence of numerous types of voltage-gated ion channels in their membrane. The distribution patterns of these ion channels are often characteristic for certain types of dendrites, neurons, or brain regions (reviewed in Migliore and Shepherd, 2002; Johnston et al., 2003; Magee, 2008; Spruston, 2008). For example, it has been demonstrated that certain ion channels have a polarized dendritic distribution, while others are distributed in a homogenous manner along the apical dendrite of pyramidal neurons (Lai and Jan, 2006). The combination of ion channels expressed by a neuron, as well as their dendritic distribution may serve as a molecular dendritic signature of a particular cell-type and assist in cell classification studies. Such information is necessary for the generation of accurate circuit maps, for the understanding of dendritic computation processes and for visualizing changes in ion channel expression during plasticity or associated with certain pathologies (reviewed in Frick and Johnston, 2005; Remy et al., 2010; Shah et al., 2010; Narayanan and Johnston, 2012; Pastoll et al., 2012). Finally, this information is required for biologically realistic neuron models (e.g., Poirazi et al., 2003).

Classical approaches for directly measuring ion channel distribution rely on the use of dendritic patch-clamp recordings or electron microscopy measurements of ion channel puncta. Such approaches are painstaking, and are unsuited to surveying the entire dendritic arbor (unless combined with labor-intensive serial section analysis and reconstruction approaches). In addition, electrophysiological measurements may be precluded by the small diameter of certain dendrites. Immunohistochemical localization of ion channel subunits, combined with high-resolution light microscopy visualization (for example using confocal or two photon microscopy) offers a higher throughput alternative for mapping the distribution of ion channels subunits along a dendritic arbor. This approach uses equipment available in most imaging facilities, making it more generally accessible to the wider neuroscience community. Moreover, this quantitative information can be correlated with the underlying structure and functional measurement of the neuron under study. Such approaches are well-suited, for example, to comparative studies involving the plastic modification of ion channel distribution following activity or in disease.

A number of approaches for extracting quantitative information regarding ion channel immunofluorescence have previously been described. One such approach that attempts to link dendritic morphology to ion channel distribution involves the selection of a region of interest (ROI) along the dendritic arbor and the calculation of average pixel intensity (following background subtraction or thresholding) from this ROI (for example Atkinson and Williams, 2009; Lee et al., 2012). In a similar manner, fluorescence intensity data can be extracted from an ROI defined by an arbitrary line drawn along a dendritic structure (Kim et al., 2007). However, a shortcoming of this method is the significant operator involvement required to define the regions of interest for each dendritic section, and the loss of three-dimensional information. Furthermore, in some cases the underlying neuronal structure was inferred from the pattern of ion channel staining thus limiting this approach to situations in which the channel is present and readily detectable along the entire length of the dendritic arbor. An alternative approach, described by Ballou et al. (2006) permits the 3D reconstruction and mapping of ion channel subunits along the dendritic axis. However, it requires significant manual intervention to set the threshold for segmentation analysis and subsequent reconstruction.

Here we describe a suite of programs for semi-automated reconstruction of dendritic arbors and the analysis of fluorescence intensity associated with a given dendritic structure. To aid the reconstruction of dendritic structure, neurons are labeled with a volume-filling fluorescent marker. Neurons are then fixed, immunostained to permit ion channel detection, and imaged using laser scanning confocal microscopy (LSCM). Confocal stacks corresponding to the volume-filling morphological marker are then subjected to image enhancement, segmentation, and skeletonization to permit the graphical reconstruction of the salient structural features of the neuron's dendrites. These processing steps take place in a semi-automatic manner with the operator required merely to supply information about the resolution of the data and provide coordinates for a seed-point for dendritic reconstruction. The resulting midline-based skeleton is then used as a reference point to map fluorescent intensity data corresponding to the signal derived from immunofluorescent detection of ion channel subunit puncta. The output from this analysis is an Igor Pro text-file, permitting further processing of numerical data, as well as a three-dimensional fluorescence intensity map that can be visualized using Amira software. This approach has numerous advantages over alternative quantitation approaches, notably, significantly reduced operator involvement, no necessity to designate arbitary ROIs and, most importantly, the ability to correlate fluorescent intensity data with structural features.

## Materials and methods

### Sample preparation

#### Animals

All experimental procedures were performed in compliance with European Union and French law, under authorization from the French Ministry of Agriculture and Fisheries (A33 10 036). Timed pregnant female C57Bl/6J mice were purchased from Charles River Laboratories or Janvier SAS (France) and maintained in a conventional animal facility prior to sacrifice.

#### Neocortical neuron culture

Unless otherwise stated, all cell culture reagents were purchased from Life Technologies (Saint Aubin, France). Dissociated mixed neuronal cultures were prepared from E17.5 C57Bl/6J mouse embryos using standard protocols. Briefly, neocortices were dissected in ice-cold dissection buffer (1 × PBS, pH 7.3, supplemented with 0.03 M Hepes and 0.33 M Glucose), digested in prewarmed 0.5% trypsin-EDTA for 8 min at 37°C, rinsed two times in 1 × PBS supplemented with 10% FBS and one time in neurobasal media and resuspended in plating media (neurobasal media complemented with 0.5 mM GlutaMAX, 1 × B27 supplement, and 50 units/ml penicillin-streptomycin). Neurons were dissociated by gentle trituration with a fire-polished glass pipette and the resulting suspension filtered through a 70 mm cell strainer (BD Biosciences). Neurons were plated at a density of 350,000 cells per 60 mm diameter dish on pre-prepared sterile, acid-etched, poly-D-lysine (Sigma)-coated cover slips (15 or 18 mm diameter, thickness 0.17 mm ± 0.01, Hecht-Assistant). After 24 h, half the plating media was exchanged and replaced with fresh plating media supplemented with 2.5% FBS. After 4 days *in vitro* (DIV) half the media was exchanged and replaced with plating media containing 4 mM cytosine-β-D-arabinoside (Sigma). Half the media was exchanged with regular plating media every 4 days thereafter. Three days before harvest, cells were infected with CVS-G pseudotyped glycoprotein-deleted rabies virus expressing mCherry or eGFP (SAD ΔG eGFP(CVS-G) or SAD ΔG mCherry (CVS-G); ~10^3^ infectious particles per ml of media).

#### Immunofluorescent staining of cultured neocortical neurons

Cultured neocortical neurons (14–16 DIV) were fixed and stained with monoclonal antibodies (NeuroMAB) against Kv2.1, Cav1.3, and Cav1.2, and Alexa-conjugated secondary antibodies (Life Technologies). eGFP or mCherry labeling of neuronal structure was amplified using polyclonal antibodies against GFP (Life Technologies) or DsRed (Clontech) and complementary Alexa-conjugated secondary antibodies. Full details of fixation, antibodies, and immunostaining procedures are described in Supplementary Methods.

### Image acquisition and processing

#### Image acquisition

High-resolution confocal image stacks were acquired using a Leica TCS SP5 laser scanning microscope equipped with Argon 488 nm-, DPSS 561 nm-, and He-Ne 594 nm lasers, and a 63× (NA 1.4) oil immersion objective. Confocal images were acquired from immunofluorescently stained cultured neocortical neurons using hybrid or conventional photomultipliers (PMT; Leica). In each sample, the two fluorophores (Alexa 488/eGFP paired with Alexa 594, or Alexa 488 paired with mCherry/Alexa 555) were imaged separately using sequential scanning to eliminate the possibility of overlapping emission. Images were obtained using near optimal Nyquist–Shannon resolution in x and y dimension. The step size of the z-stack was selected to ensure that voxel size was more or less isotropic in all three dimensions. 12-bit images were acquired at line scan frequencies of 400 Hz and a line average of 2 for morphological structures and 4 for signal related to ion channel subunits. Pinhole was set to 1 (Airy Unit).

#### Image processing

***Custom-written analysis software.*** Several custom-written programs were used for the image processing. Image filtering and segmentation were performed using *Anisofilter* and *Segment*. Skeletonization was performed with *DendriteTracker*, and the *DendriteTracker* and *DendriteAnalyser* used for the tracking and analysis of fluorescent intensity signals in 3D space. These programs were run using the UNIX emulator, Terminal.

***Conversion, filtering, and segmentation.*** Confocal image stacks were saved as 8-bit format multilayer tif-files. The native Leica image stacks were imported into ImageJ (v1.44o; http://imagej.nih.gov/ij), converted to 8-bit format and saved as separate multilayer tif-files for each channel. Subsequent image processing steps were performed on either non-deconvolved or deconvolved 8-bit multilayer image data using a Mac Pro 2.8 GHz Quad-core Intel Xeon computer equipped with 18 GB RAM, and running MacOS 10.6. Multilayer tif-files corresponding to the morphological signal (eGFP or mCherry) were subjected to two rounds of filtering and segmentation using the custom-written software *Anisofilter* and *Segment* as described previously (Broser et al., 2004; Oberlaender et al., 2007).

***Skeletonization.*** Dendritic skeletons (approximate midlines) were reconstructed from the aforementioned-segmented images using the custom-written program *DendriteTracker*, described previously in Oberlaender et al. (2007), Broser et al. (2008). Briefly, the user starts *DendriteTracker* utilizing the segmented image as input and image size (in μm) and cell body coordinates (x, y, and z, pixel units) as parameters. The first step of the program is a raster-to-vector image conversion. The resulting vectors, hereafter referred to as compartments, contain the 3D coordinates of the foreground voxels corresponding to the neuron structure. These coordinates are subsequently used as a reference point for the generation of data sets corresponding to dendrite radius and fluorescent intensity (see below). The next step is a vector image-based midline extraction. We used the template-matching algorithm described by Jonker (2002) to calculate the skeleton. Dendritic end-points were established by searching for the distant-most compartment with respect to the cell body position. The resulting skeleton was converted and saved as a Neuron hoc-file (Hines and Carnevale, 2001).

***Ion channel signal quantification.*** Quantitation of dendritic ion channel signal was performed using the custom-written program *DendriteAnalyser*. *DendriteAnalyser* was started from the command line with the hoc geometry file and the native (or deconvolved) multi-layer tif-file corresponding to the ion channel signal as input. Since the original datasets corresponding to both morphological- and ion channel signal were generated during the same imaging session, the 3D coordinates derived above match the same topographical location in the ion channel image file. As described above, the geometry of the neuron is represented in a graphical structure in which the edges represent the linear dendritic structures and the nodes represent the bifurcation between the dendrites or cell body. Each dendritic segment is represented as a list of compartments with each compartment containing a vector associated with the original xyz position in the imaging stack. *DendriteAnalyser* then scans over the graphical structure, selecting one dendrite at a time. Next, the program selects one compartment after the other from the list and registers the corresponding signal in the native ion channel file at each given vector position. As a result of this analysis, a number of datasets are calculated, namely: (1) A single value at the position of the compartment (point value). (2) The mean value; the mean is calculated by a spherical volumetric window moving along the skeleton, and the sphere (diameter of 6 μm) is scaled by the actual voxel dimensions. (3) The maximum value in the compartment around the sphere. (4) The radius of the dendrite derived from the morphological channel is reported in a separate data set as described above. The calculated values for each compartment (voxel position) of a dendrite are stored and written to disk as a txt file. In addition, a file combining the three-dimensional cell geometry and the measured values was created. The geometry was visualized using the software Amira (VSG, France).

***Image presentation.*** Native (unprocessed) and processed (filtered, segmented) images were imported into ImageJ and a z-projection prepared from maximum signal intensity derived from the relevant focal planes. Maximum projection images were then imported into Adobe Photoshop CS3 and adjustments for brightness, contrast, and color levels performed for illustrative purposes only. Images were extracted from the Amira visualization software using the screen capture function. Images were then imported into Adobe Photoshop CS3 and a ROI selected for final presentation.

#### Additional information and resources

A detailed user protocol and instructions for downloading the algorithms described herein can be found at the following links: http://www.neurocentre-magendie.fr/NCM_Pages/Equipes/eq_frick/UK_equipe_frick.php and drbroser.org.

## Results

### Fluorescent labeling of neuronal morphology

**Figure 1:**
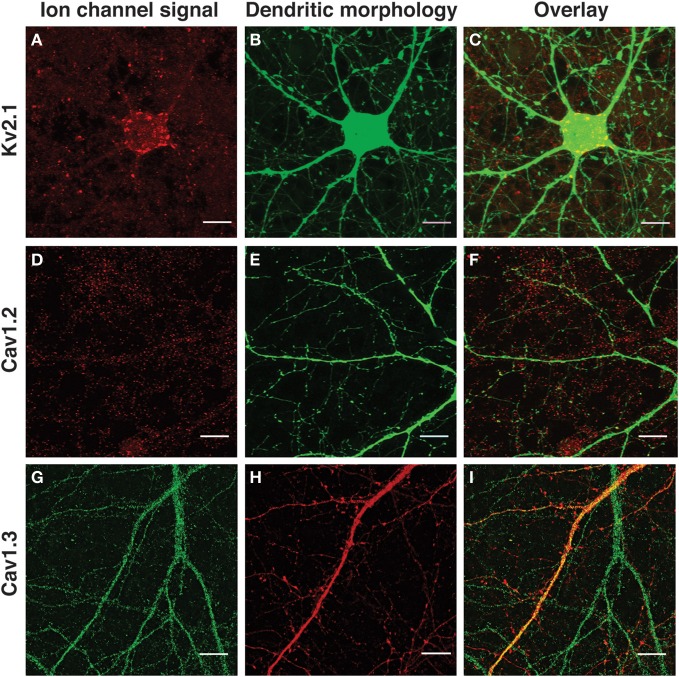
**Immunological detection of voltage-gated ion channel subunits in SAD ΔG-labeled cultured neocortical neurons.** **(A,D,G)** Immunofluorescence detection of endogenous ion channel signal. **(B,E,H)** Morphological labeling of neuronal structure (mCherry or eGFP). **(C,F,I)** Overlay of ion channel signal and neuronal morphology. **(A,C)** Kv2.1; **(D,F)** Cav1.2; **(G,I)** Cav1.3. Images are a zoom of a selected area of the same neurons presented in Figures 4A,B, 5A, and 6A–C. All images are Z-projections calculated from maximum intensity signals derived from all focal planes of the image stack in which ion channel or morphological signal appears. Scale bar, 10 μm.

Neuronal structure was labeled by infecting cultured neurons with a pseudotyped single-cycle rabies virus vector [SAD ΔG (CVS-G); Etessami et al., 2000; for recent review see Ginger et al., 2013] expressing eGFP or mCherry. Virus titers were adjusted to ensure medium-to-sparse labeling of the neuronal population to facilitate subsequent skeletonization and quantification steps. High-level fluorescent protein expression was observed 3 days after infection (Figures 1B,E,H). Fluorescent intensity was further enhanced by the use of an antibody against GFP or DsRed and subsequent use of an appropriate Alexa 488- or Alexa 555-conjugated secondary antibody. This amplification step was not essential, but enabled us to use low laser power for fluorophore excitation and thus avoid photobleaching as a result of the high rate of sampling in the z-dimension.

### Immunological detection of ion channel subunits

SAD ΔG (CVS-G)-labeled neocortical neurons were fixed using previously published protocols (Lim et al., 2000; Zhang et al., 2006)—adapted in each case to the ion channel subunit under study. Ion channel subunits were detected using monoclonal antibodies that have been characterized in previous studies (Misonou et al., 2006; Fossat et al., 2010; Fu et al., 2011; Schierberl et al., 2011; Brandao et al., 2012). Appropriate negative controls (in which either the primary antibody or secondary antibody were omitted from the immunostaining procedure) were included in this analysis to ensure the specificity of staining for each antibody (data not shown). In each case, the pattern of staining obtained was compared to staining previously demonstrated in neocortical slices (Hell et al., 1993; Guan et al., 2007; Marshall et al., 2011) and in cultured hippocampal neurons (Lim et al., 2000; Misonou et al., 2004; Obermair et al., 2004; Zhang et al., 2006; Kim et al., 2007). For our analysis, we choose to label and analyze one subunit of a delayed K^+^ channel (Kv2.1) and two subunits of L-type Ca^2+^ channels (Cav1.2 and Cav1.3).

Kv2.1 (a member of the *Shab* family of K^+^ channels and a major subunit contributing to delayed rectifier K^+^ current) has as characteristic, highly polarized pattern of expression within neurons of the rodent forebrain (reviewed in Vacher et al., 2008). In our own experiments, Kv2.1 immunostaining was visualized as large, well-separated, prominent clusters associated with the cell body and proximal dendritic structures (Figures 1A–C), reminiscent of the pattern of expression previously reported for hippocampal neurons (Lee et al., 2004; Misonou et al., 2004). Sparse, faintly stained, well-separated Kv2.1 punctae were also detected along the length of the dendritic shaft (consistent with EM data; Du et al., 1998).

Cav1.2 and Cav1.3 correspond to the a1C and a1D subunits, respectively, of L-type calcium channels. Both subunits were distributed in a punctate manner along the entire length of the dendritic arbor with no apparent gradient with respect to distal or proximal dendritic location or bias for large or small dendrites (Figures 1D–I). A similar pattern of expression has previously been reported for Cav1.2 and Cav1.3 in cultured hippocampal neurons (Obermair et al., 2004; Zhang et al., 2005, 2006). Cav1.2 expression appeared to be more clustered in nature than Cav1.3; the latter was observed as small, finely distributed punctae along the dendritic arbor. In these three cases, the pattern of ion channel subunit expression was in good agreement with previously published literature and provided a distinct and characteristic set of examples to test our quantification algorithm.

### Image acquisition

High-resolution stacks were obtained as described in Materials and Methods and using many of the recommendations for confocal scanning set out in Wimmer and Möller (2010). In each case, PMT settings were carefully selected using the quick look-up-table (QLUT) function to ensure that the resulting image represented the entire dynamic range available. Test images were then captured in both channels (see Methods) using the same line average and other setting as the final image and visually inspected in QLUT mode. Test images were then imported into ImageJ and manually inspected by examining the fluorescent intensity (0–4096, corresponding to a 12-bit image) along a dendritic structure. The cell body area (which was intensely labeled by eGFP or mCherry fluorescence) was excluded from this initial analysis, as it was important that dendritic structures were imaged with a sufficient dynamic range to allow subsequent reconstruction. Cell body structures were thus saturated in this process.

PMT acquisition windows were carefully set to limit the possibility of capturing signal derived from the excitation of non-target fluorophores. This was less of a problem for neurons labeled with eGFP/Alexa 488 and Alexa 594 because the excitation/emission spectra of these fluorophores are well-segregated. However, there is a significant overlap of the tail region of the excitation spectrum for Alexa 555 and the excitation spectrum for Alexa 488. Controls were therefore performed in which we set the acquisition window for one fluorophore and substituted the excitation laser of the other fluorophore (data not shown). These controls were necessary to ensure that the signal relating to ion channel immunofluorescence was specific for the ion channel and not due to “bleed-through” from the morphological marker or vice versa.

Images were acquired with settings affording the best approximation of Nyquist-Shannon sampling rates. These settings were defined by the numerical aperture of the objective and by an idealized average light wavelength of 532 nm. Image stacks were acquired using 2048 × 2048 format, an optical zoom of 1.5 and z-step of 0.08 μm resulting in a voxel size of 80.13 × 80.13 × 83.92 nm. The isotropic dimensions in x, y, and z were important for the subsequent analysis of fluorescent intensity data obtained from *DendriteAnalyser*. It is important to note that this resulted in oversampling in the z-dimension, however we were able to avoid problems associated with photobleaching by using a hybrid PMT for the ion channel signal and by keeping the corresponding laser power to a very low level. One shortcoming of these acquisition parameters was the long time required to acquire one image stack (i.e., ~30 min for the acquisition of 90 z-stacks) even when employing bi-directional scanning. Images were typically acquired with line-scan speeds of 400 Hz to allow high quality image acquisition. Higher scanning speeds were tested, including the use of a resonance scanner (scanning at 8000 Hz) to enable more rapid acquisition of tiled images, but this resulted in loss of image quality (data not shown). To reduce the effects of oversampling in the z-dimension and to diminish the long acquisition time, one can reduce image size to 1024 × 1024 format and increase the z-stack size to 0.17 μm. This has the effect of increasing the voxel size in all three dimensions. The choice of the appropriate format has to be empirically tested in each case.

### Image processing

**Figure 2:**
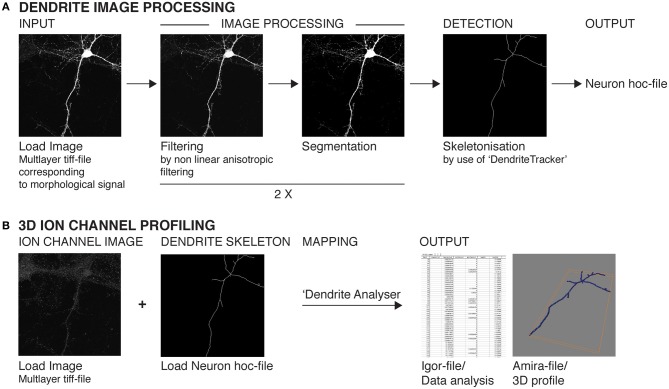
**Schematic representation of the major steps involved in image processing, skeletonization, and ion channel profiling analysis. (A)** Images stacks pertaining to neuronal morphology data (eGFP or mCherry expression) are subjected to two sequential rounds of filtering and segmentation analysis using custom-written algorithms *Anisofilter* and *Segment*. Skeletonization is performed using *DendriteTracker*. The resulting output (in hoc format) can be visualized using Neuron. **(B)** The image stacks corresponding to ion channel signal are then subjected to 3D profiling analysis using the custom-written algorithm *DendriteAnalyser*. The dendritic skeleton (Neuron hoc-file) serves as reference for the 3D coordinates for fluorescent intensity calculations. The resulting analysis is written to Amira mesh and Igor Pro wave format. All confocal images stacks are converted to multi-layer tiff-files in 8-bit format prior to analysis.

The basic schema for image processing is described in Figure 2. Raw imaging data typically consisted of ~40–95 optical sections containing data from two channels at 12-bit depth. These image stacks were imported into ImageJ, converted to 8-bit format to facilitate subsequent image processing and each channel saved separately as a multilayer tif-file. Morphological data (resulting from eGFP or mCherry signal) was then treated separately from the ion channel data for the subsequent processing steps (Figure 2A). Data was first subjected to a filtering procedure using a non-linear anisotropic diffusion filter (Broser et al., 2004). The filtered data was subsequently segmented and then the filter and segmentation steps repeated as described in Broser et al. (2004). Morphological data was then subjected to skeletonization using the custom-written reconstruction algorithm *DendriteTracker*. This algorithm requires the user to provide image size and cell body coordinates, which provide a seed-point for dendrite skeletonization. Where no cell body is present, a proxy location along the dendritic arbor must be given to provide an equivalent seed-point. Image size was obtained from the descriptive text file extracted from the original Leica lif-file. Cell body coordinates were derived by inspecting the filtered, segmented data in ImageJ. The output from this initial processing was a Neuron hoc-file. This file was inspected in Neuron—selecting “distributed mechanisms” and then “shape names” to display the skeletonized structure. Clicking on a selected segment of a dendrite revealed the name associated with this segment and its length in microns. This data was retained for subsequent analysis using *DendriteAnalysis* and *Igor*.

**Figure 3:**
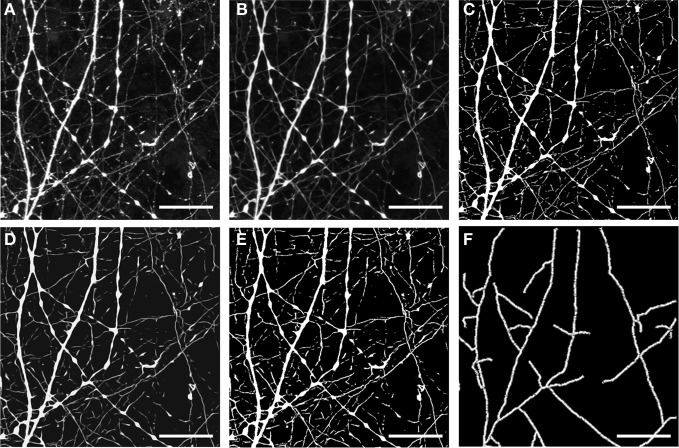
**Representative example of outcome of the different steps in dendrite image processing. (A)** Raw data—image stack corresponding to morphological structure (in this case resulting from eGFP expression). **(B)** The same image stack following filtering; **(C)** filtering and segmentation; **(D)** filtering and segmentation, followed by a second round of filtering; **(E)** two sequential rounds of filtering and segmentation. Images shown in **(A–E)** are z-projections calculated from all focal planes. **(F)** Image after skeletonization of resulting segments [from data shown in **(E)**]. Scale bar, 10 μm.

**Figure 4:**
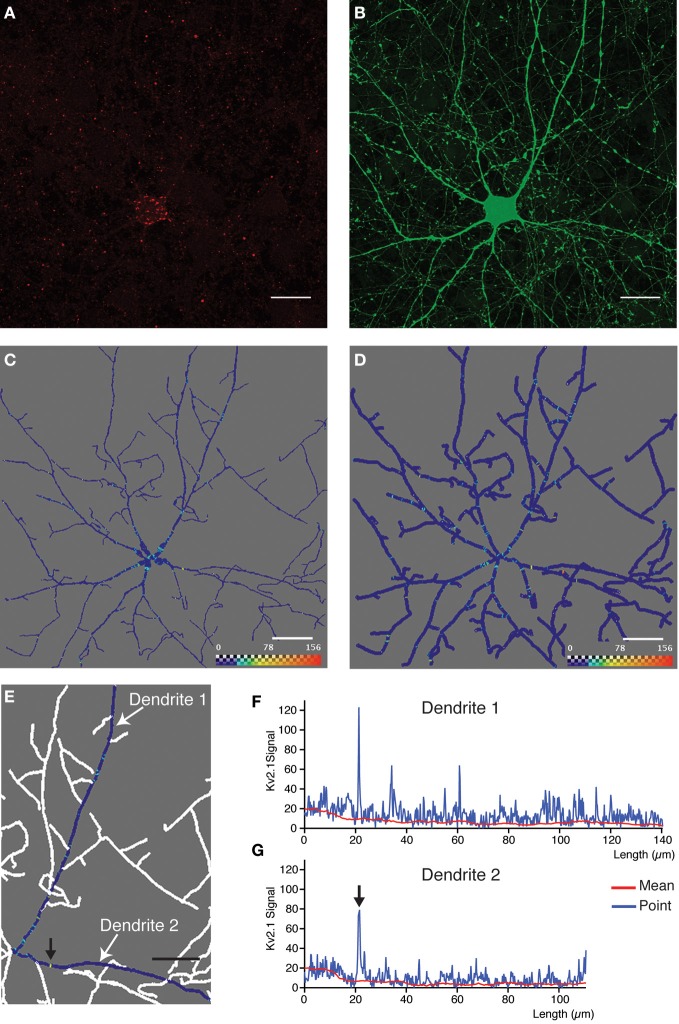
**Quantitative analysis and 3D profiling of endogenous Kv2.1 signal in cultured neocortical neurons. (A,B)** Image stacks corresponding to endogenous Kv2.1 signal **(A)** and morphological label (eGFP expression) **(B)**. Images are z-projections derived from maximum signal intensity across 36 focal planes generated from data that has been subjected to 2 rounds of blind deconvolution. **(C)** Two-dimensional snapshot taken from Amira viewer window showing neuronal reconstruction overlaid with 3D ion channel profile derived from point fluorescence values calculated along the dendrite skeleton. The color map has been adjusted to best represent, on a graphical level, the predominant ion channel clusters. Scale bar: 20 μm. In this case, the skeleton is represented as a true morphological skeleton (incorporating information about the radius of the dendrite). **(D)** Two-dimensional snapshot taken from Amira viewer window (same dataset as in **C**) showing reconstructed skeleton (represented as a filament). **(E)** Magnification of a section of the reconstructed skeleton, showing 3D ion channel profile associated with two individual dendrites (arbitrarily named dendrite 1 and dendrite 2). Here, point fluorescence's values are presented by means of the color map function. The color map has been adjusted to best represent, on a graphical level, the predominant ion channel clusters. **(F,G)** Fluorescence intensity plotted as a function of dendritic length for the same dendrites. Data is presented as the point fluorescent value as well as mean fluorescent value calculated at each point. Arrows in **(E)** and **(G)** indicate a putative ion channel cluster, clearly visible in both representations of the data. Scale bar, 20 μm.

An example of the type of results obtained from this initial image processing procedure is shown in Figure 3. In this case we selected an image stack containing a cell with numerous dendrites arranged in a “stellate”-type arrangement. The image stack also contains substantial background complexity due to overlapping neuronal processes arising from other neurons within the culture network. This example shows a magnified section of processed data from the same image stack as that presented in Figure 4B. Comparison of the raw data (presented in Figure 3A) with data following one round of anisotropic filtering (Figure 3B) shows the immediate enhancement in signal obtained as a result of this filtering step. In this panel, two prominent dendritic structures diverging from a single branch point are clearly visible, in additional to a number of thinner beaded structures (most likely axons) as well as numerous small processes. Following two rounds of filtering and segmentation (Figures 3C–E), the two major dendritic structures appeared thicker, whereas the numerous small processes had become more fragmented and were not reconstructed in the subsequent skeletonization process (Figure 3F). Thus, this initial image processing procedure has the effect of enhancing the recovery and reconstruction of certain structures and at the same time of removing noise due to smaller, non-related structures.

**Figure 5:**
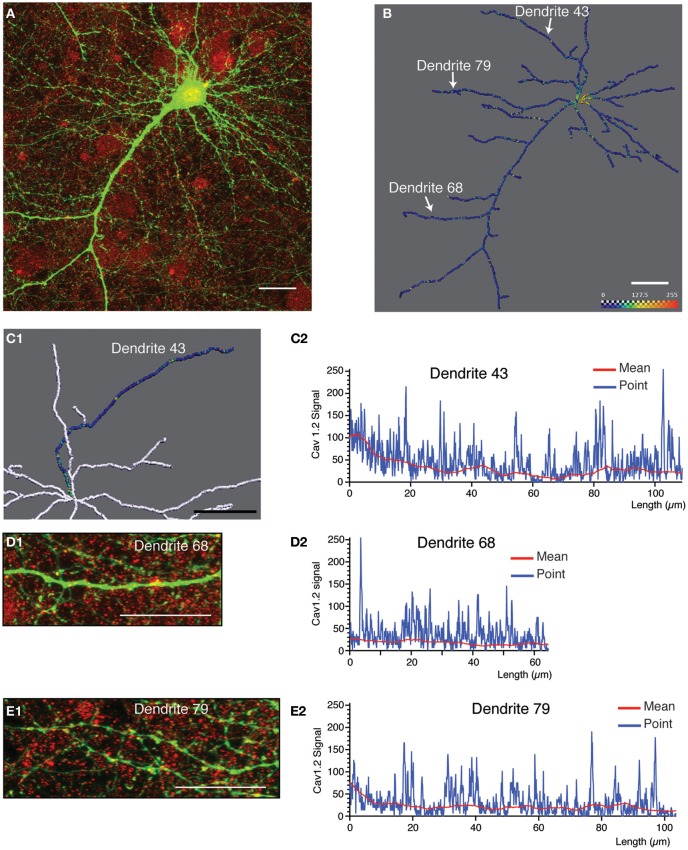
**Quantitative analysis and 3D profiling of endogenous Cav1.2 signal in cultured neocortical neurons. (A)** Image stack corresponding to endogenous Cav1.2 signal (red) overlaid with neuronal morphology (labeled by eGFP expression, green). Image is a z-projection derived from maximum signal intensity across 65 focal planes. **(B)** Amira image showing reconstructed dendritic skeleton (represented as a filament) overlaid with 3D ion channel profile derived from point fluorescence values calculated along the dendrite skeleton. The color map has been adjusted to best represent, on a graphical level, the predominant ion channel clusters. **(C1)** Magnification of a section of the reconstructed skeleton showing the ion channel profile of a selected dendrite (dendrite 43). Image has been flipped (reflected along vertical axis) to better correspond to the data shown in **(C2)**. **(C2)** Fluorescent intensity plots for the same dendrite as in **(C1)**. Data is presented as the point fluorescent value as well as mean fluorescent value calculated at each point. **(D1,D2,E1,E2)** Analysis for two other selected dendrites (dendrite 68 and dendrite 79). The position of these dendrites within the neuronal skeleton is indicated in panel **(B)**. **(D1,E1)** Region of interest extracted from the same native image stack as **(A)** showing endogenous Cav1.2 signal (red) overlaid with morphological label (eGFP, green) for two selected dendrites. Images are z-projections derived from maximum intensity over selected focal planes. **(D2,E2)** Fluorescent intensity plots for the same dendrites. Data is presented as the point fluorescent value and mean fluorescent value calculated at each point. Scale bar, 20 μm.

**Figure 6:**
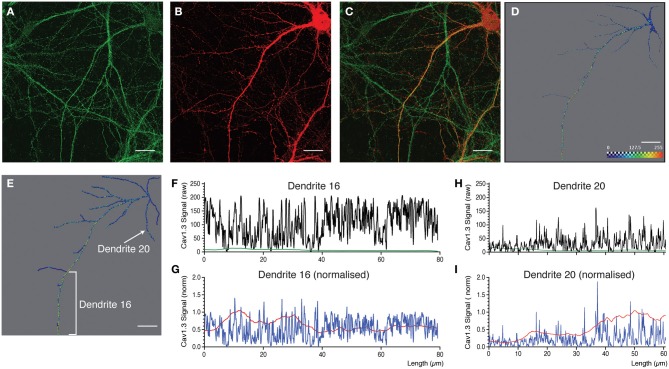
**Quantitative analysis and 3D profiling of endogenous Cav1.3 signal in cultured neocortical neurons. (A,B)** Image stacks corresponding to endogenous Cav1.3 signal **(A)** and morphological label (mCherry expression) **(B)**. **(C)** Overlay of images in (**A** and **B**). Images are z-projections derived from maximum signal intensity across 24 focal planes. **(D,E)** Amira images showing reconstructed dendritic skeleton overlaid with 3D ion channel profile derived from point fluorescence values calculated along the dendrite skeleton. **(D)** Morphological skeleton with dendritic dimensions representing the radius of the true dendrite structure. **(E)** Filament map. **(F,G,H,I)** Fluorescent intensity plots for two selected dendrites (indicated in panel **E**). Data is presented as the point fluorescent value and mean fluorescent value calculated at each point. **(F,H)** Raw data. **(G,I)** Data normalized to fluorescent intensity data obtained from applying the same analysis to the morphological label. Scale bar, 20 μm.

The resulting reconstructed skeleton nevertheless still retained a number of non-dendritic structures due to the high efficiency of morphological labeling and the complexity of the network of neuronal processes in this particular example. This issue can be effectively dealt with in the subsequent analysis step, in which the *DendriteAnalyser* algorithm provides the user with the option of manually entering a sequence of dendritic segments forming part of a larger dendritic structure. This option provides a means of selecting out unwanted information. We also tested whether deconvolution of the image stack prior to 8-bit conversion and initial filtering improves the reconstruction (data not shown). Although in some cases deconvolution resulted in a cleaner reconstruction (e.g., for the data presented in Figure 3A, in other cases it actually diminished the signal intensity to the point that reconstruction was impaired (e.g., for data presented in Figures 5, 6). The decision whether or not to deconvolve the raw image stack needs to be empirically determined in each particular example by the user. It is important to note, however, that once this and the aforementioned imaging parameters are established for an experiment, they must be kept constant for all samples pertaining that experiment, enabling comparison of different experimental conditions.

### 3D ion channel profiling

The next step in the image analysis procedure (exemplified in Figure 2B) requires the multilayer tif-file corresponding to ion channel signal and Neuron hoc-file produced by the *DendriteTracker* algorithm. These files provide the input for *DendriteAnalyser*. The output is an Igor wave file containing fluorescence intensity and geometric data for each dendrite or dendritic segment and an *Amira* file permitting 3D visualization of this analysis. The results of this type of analysis for three different ion channel subunits are presented in Figures 4, 5 and 6. In the first example, the dendritic morphology data is taken from the same complex example presented in part in Figure 3. In this case, analysis was performed on data derived from deconvolution of the native Leica lif-file. The *DendriteAnalyser* output results in several datasets (as described in Materials and Methods). Here, we have chosen to present the point fluorescence values plotted in 3D space along the entire dendritic skeleton (Figures 4C,D). In the first case (Figure 4C) the skeleton is presented as a realistic structure integrating information from the dataset pertaining to dendrite radius. In the second case (Figure 4D), the skeleton is represented as a filament, aiding the visualization of color map data. Note that fluorescent intensity values corresponding to the first 5–7 μm of the “dendritic” segment actually correspond to the soma because the center point of the cell body is used as a seed-point for reconstruction. This data should be excluded from any subsequent analysis.

The data from two selected dendrites are presented in Figures 4E–G. The data for an individual dendrite were obtained by entering a sequence of “dendritic segments” during the interactive phase of the analysis (as described above). In Figures 4F,G the data is presented as both point fluorescence intensity as well as mean fluorescence intensity within a sphere, plotted in relation to dendrite length. In such examples in which the signal is highly punctate and membrane-associated, the point fluorescence value is likely the best measure for identifying clusters. This analysis is in good agreement with the pattern of expression observed at the visual level, with the majority of prominently stained Kv2.1 clusters located proximal to the cell body. Note that the prominently stained cluster indicated by an arrow on the Amira image (Figure 4E) is also apparent on the fluorescent intensity plot (Figure 4G). Smaller, less intensely stained clusters (barely discernable by visual inspection of the image) are also readily detectable by this type of analysis, thus offering a greater degree of precision than previous afforded by visual interpretation of imaging data.

For the second example, we selected an image containing more simple morphological information (indicated by eGFP expression, green; Figure 5A). Shown is a typical pyramidal neuron with an apical dendrite that has one major branch-point, and a number of smaller basal dendrites and other processes (most likely axons). This neuron was immunostained with a monoclonal antibody against the L-type calcium channel subunit, Cav1.2 (Figure 5A, red). Morphological reconstruction of the neuron resulted in detection of most of the basal dendrites, and side branches in addition to the main apical dendrite (Figure 5B). The skeleton is represented as a simple filament, and the fluorescence intensity data relating to point fluorescence values along the dendritic skeleton are presented in form of a color map image. Cav1.2 immunolabeling exhibited a highly punctate pattern of dendritic expression (Figures 5A,B). The punctae were prominently stained, but nonetheless smaller than those observed for Kv2.1 and appear to be relatively evenly spaced along the dendritic arbor (Figures 5D1,E1) in contrast to the characteristic staining for Kv2.1. Intense Cav1.2 immunolabeling was also detected in the cell body. Thus, as discussed above (Figure 4) the first 5–7 μm of the reconstructed dendrite should be excluded from the analysis. By inspecting the fluorescence intensity plots pertaining to the point fluorescence value for several individual dendrites (Figures 5C2,D2,E2) we noted several elements of the staining pattern: Firstly, a base-line level of fluorescence ranging from 0 to about 40 levels of gray; secondly, a medium-intensity staining (ranging from 40 to 120 levels of gray) relating to faintly stained clusters; and lastly, prominently stained clusters (with fluorescent intensity values between 120 and 255) that are readily observable by eye. As the analysis corresponds to raw data, intensity values representing the entire dynamic range are discernable. The color map in Figures 5B,C was adjusted to better represent these differences in staining intensity. Figure 5C1 shows a section of the reconstructed skeleton (shown predominantly in white) with the three-dimensional fluorescent intensity profile of one dendrite of interest (Dendrite 43) shown in color. A number of intensely stained Cav1.2 clusters are readily discernable in both this color map profile, as well as in the corresponding fluorescent intensity plot (Figure 5C2).

For the last example (Figure 6) we selected a neuron with a prominent apical dendrite and numerous side branches. Cav1.3 immunostaining is presented in Figure 6A, and the morphological label (mCherry expression) in Figure 6B. In Figures 6D,E, the corresponding reconstructed dendritic skeleton is presented, together with the ion channel profile derived from point fluorescence intensity values. In Figure 6D the skeleton is presented as a morphologically realistic reconstruction, while a filament is presented in Figure 6E. As stated above, Cav1.3 immunostaining resulted in fine-grained punctae that extend along the entire dendritic length (Figures 6A,C). The main apical dendrite was intensely immunostained (see selected dendritic segment, Dendrite 16, as an example, Figures 6D,F). However, basal dendrites and side branches showed weaker immunostaining with more discrete punctae (see Dendrite 20, Figure 6H as an example). This analysis shows that it is possible to distinguish different patterns of staining, even within the same neuron. In the aforementioned analyses, data is presented as raw fluorescent intensity values related to individual points along the dendrite or as mean fluorescent values derived from raw data. In certain situation (see discussion) it might be advantageous to present normalized values instead of raw data. To do this, we repeated the analysis, using raw, unfiltered morphological data as the input for *DendriteAnalyser*. This generated a compatible set of fluorescent intensity values for the morphological signal. These values were then used as a means of normalizing the fluorescent intensity derived from the ion channel signal. The results of these analyses (Figures 6G,I) show that normalizing can enhance certain information. For example the punctae become more pronounced for Dendrite 20 (Figure 6I). Moreover, the mean fluorescent value becomes more predictive for the overall change in fluorescent values along the dendrite and may in certain situations become a more useful value than point fluorescent values.

## Discussion

Quantitative analysis of dendritic protein expression/localization, in combination with detailed morphological analysis of dendritic structure can reveal important information regarding the identity of a neuron and aid predictions of its function within a circuit. Traditional approaches for this type of analysis (EM/direct dendritic recordings) are time-consuming, highly specialized, and poorly adapted to more rapid screening processes necessary for detecting changes associated with altered physiological state or disease. Whilst the aforementioned techniques remain the gold standard for this type of analysis, light microscopy approaches offer a higher-throughput alternative, permitting the screening of a statistically appropriate number of samples required to detect such changes.

### Requirements/parameters

The success of our approach depends on five main requirements: (1) The specificity of immunological protocols; (2) the choice of appropriate imaging strategies; (3) appropriate morphological markers to permit the neuroanatomical identification of target cell-type and dendrite geometry; (4) targeted computational methods that permit the unbiased, semi-automated quantitation of ion channel signal in three-dimensional space; (5) methods for normalization to permit the unbiased comparison of samples. The first point has been discussed in detail previously (Rhodes and Trimmer, 2006; Lorincz and Nusser, 2008). We would like to re-iterate the importance of validating each immunological protocol for a given situation (e.g., cell-type, tissue etc.). Where possible, it is also advisable to ensure that the pattern of ion channel localization is in accordance with that obtained by EM data (Nusser, 2012).

Here we present a method that can be readily applied to LSCM data. The method should work equally well with imaging data obtained with two-photon microscopy, spinning disk confocal microscopy, or array tomography, providing the same principles for sample preparation and image acquisition are adhered to. For each imaging system, the individual settings may need to be adapted for each system/situation. The most important parameters to respect are the need to preserve isotropic dimensions, to use the full dynamic range of the PMT (or CCD camera), to define settings that permit good spectral separation and the use of an objective with a high numerical aperture. Equally, the decision whether or not to deconvolve before analysis is something that must be empirically determined by each user. The anisotropic diffusion filtering employed in the treatment of the resulting image has the effect of smoothing along neuronal processes and repairing any gaps in the structure of the dendrite (Broser et al., 2004). It is nonetheless important to carefully select upper and lower z-limits to ensure that all of the structure is present in the resulting image stack because this processing step cannot repair missing data.

In the examples presented above, morphological information was obtained from virus-mediated expression of a volume-filling marker (eGFP or mCherry). In our approach we took advantage of the neurotropic nature and high level of expression permitted by the use of glycoprotein-deleted rabies virus (Etessami et al., 2000; reviewed in Ginger et al., 2013). However, the method could be equally adapted to other labeling methods such as biocytin filling, plasmid transfection, or the use of a transgenic mouse line expression a fluorescent marker in a cell type-dependent manner (e.g., Feng et al., 2000; Gong et al., 2003). The major requirement is that the marker is homogenously distributed throughout the neuron and not excluded from certain structures. In the aforementioned analysis, the distinction between dendrite and axon was based on visual inspection and characteristic morphological features (for example the presence of prominent boutons on axonal processes, see Figure 3 for an illustration of this point). For other cell-types/situations, this distinction may not be as obvious and it may be necessary to explore the use of an additional morphological marker such as MAP2 to verify the identity of a structure. Our approach permits the skeletonization/reconstruction of most dendritic structures as well as providing information regarding ion channel distribution in 3D space. In addition, this method is largely automated (see below). The only decision on the part of the operator concerns the choice of the cell body center-point. Thus, the method is largely without risk of operator bias. Lastly, in certain cases (one example of which is presented in Figure 6) it may sometimes be advantageous to normalize the ion channel signal to gain a better idea of its overall distribution along a dendrite. This method has the advantage that the normalizing signal is an intrinsic part of the general methodological approach. To extract fluorescence intensity data associated with the morphological label, it suffices to repeat the *DendriteAnalyser* procedure for the image stack corresponding to eGFP or mCherry signal (or equivalent marker) and to incorporate the resulting data into the downstream analysis in *Igor Pro*. The ability to normalize the fluorescence intensity data related to ion channel expression permits the comparison of different physiological states. It should be noted, however, that such approaches require the user to explicitly validate the chosen morphology marker and to show that its expression is unaltered by physiological state. The ability to normalize also provides the possibility of correcting for loss of fluorescent signal due to, for example, shadowing effects resulting from factors such as the presence of a blood vessel in tissue. Although it has not explicitly been demonstrated here, this approach should also be adaptable to applications involving live or fixed tissue.

### Effort/time considerations

As mentioned above, the method is semi-automated and requires little involvement on the part of the operator. The largest investment of personnel effort is associated with establishing the initial imaging parameters. However, once established for a given channel/cell-type, these parameters should be saved and all subsequent image acquisition performed with the same settings. The processing times and operator effort for all subsequent steps are presented in Table A1. For the most part all that the operator is required to do is to launch the procedure and provide the correct input and output file name. The last two steps are of variable length, depending on the complexity of the cell, number of segments, and whether or not the operator chooses to individually analyze specific dendritic branches.

### Limitations/constraints

Our method reconstructs the majority of dendritic processes and provides a measurement of fluorescent intensity levels with respect to dendrite length. This method permits the analysis of relative expression levels of dendritic proteins and its correlation with different aspects of dendritic morphology (e.g., distance from soma or branch point, type of dendrite). It thus enables an examination of dendritic protein distribution or density as a function of dendritic morphology. However, it does not provide quantitative measurement of the number of molecules or size of the clusters beyond the resolution of the imaging setup. EM or quantitative super-resolution microscopy approaches are better suited for this type of analysis. In contrast, this approach allows the rapid appraisal of cell-wide and cell-type-specific patterns of expression facilitating the characterization of a “dendritic signature” for different neurons within a circuit.

This approach utilizes an automated segmentation paradigm. One disadvantage of such approaches is that the user is unable to inspect and correct any errors in the segmentation procedure as they occur. Nevertheless, it is important to point out that the anisotropic filtering step (described previously in Broser et al., 2004), which is an integral part of our image processing approach is designed to prepare the data for optimal treatment with the segmentation algorithm. As stated previously (Broser et al., 2004), the filtering process leads to improved separation of signal from noise and may actually “repair” apparent gaps in the dendritic structure. We have found that two rounds of treatment with these filtering/segmentation algorithms (as opposed to one) markedly improve the success of skeletonization using *DendriteTracker*. It is important to note that the success of this reconstruction algorithm also depends on the presence of strong morphological signal and good imaging parameters (as described in Materials and Methods). Situations in which the user does not exploit the full dynamic range of the PMT/fluorophore combination (with respect to dendritic and not cell body signal) may result in unsatisfactory reconstruction of neuronal morphology. Moreover, any pretreatment step (such as deconvolution) may also introduce its own filtering/thresholding parameters, thus diminishing dendritic signal and thereby the success of reconstruction approaches. The reconstruction approach described herein uses a vector-based midline extraction procedure to render a skeleton based on the dendrite midline. This procedure provides an accurate reference point for fluorescent intensity measurements. Its disadvantage is that the skeleton is presented as a simple wire-frame, rather than retaining real dendritic dimensions. However, this structural information is retained in the dataset related to the dendrite diameter and can be visualized in Amira (see Figure 4C and also User Manual). Altogether, our method encompasses a rapid, semi-automated method that provides sufficient morphological data to enable the characterization of the cell type under consideration.

Variations in dendritic ion channel distribution may also involve their preferential localization to sub-dendritic compartments such as spine heads/necks in addition to the main dendritic shaft. Our approach will not permit this type of analysis because the segmentation step excludes compartments that are smaller than 5 μm in length and thus spines are not reconstructed with the main dendritic branch. However, once again, this type of specialized analysis might be better suited to EM or super-resolution microscopy. In addition our method cannot be used to differentiate between cytosolic expression and that associated with the membrane. However, the use of exogenously expressed surface-specific tagged subunits or antibodies targeted to extracellular domains may overcome this problem. Lastly, although our method can be used to determine relative distributions of proteins of interest along a dendrite, it cannot be used to quantify the actual number of molecules at a given dendritic distance. As far as we are aware, the only technique that can definitively do this in both tissue and cultured neurons is EM.

### Applications

Knowledge of the sub-cellular distribution and density of a given voltage-gated ion channel is essential for understanding the role of this channel (or any protein for that matter) in neuronal information processing and storage. For many ion channels, variations in these parameters provide a way of dramatically increasing neuronal diversity in the CNS (Nusser, 2012). Unfortunately, this fundamental knowledge is lacking for most ion channels and neuron types, due to the complex dendritic and axonal morphologies of most CNS neurons. The method described herein enables the semi-quantitative analysis of ion channel distribution/density patterns across neuronal processes in three dimensions.

What kind of knowledge can one glean from this data? As mentioned above, voltage-gated ion channel distribution patterns are characteristic for specific neuron types and could therefore serve as “fingerprints” of these neurons. It will be important to understand the functional consequence of these ion channel fingerprints. For example, previous studies on the dendrites of CA1 pyramidal neurons revealed non-uniform distribution patterns of HCN and Kv4.2 ion channels along their dendrites. These distribution patterns could be linked to the role of these ion channels in regulating dendritic signaling (Hoffman et al., 1997; Magee, 1998; Lorincz et al., 2002; Frick et al., 2003). In addition to neuron-type specific patterns, certain ion channels may also display variability within a single neuron-type. For instance, Garden et al. (2008) demonstrated that HCN densities in layer 2 stellate cells of the entorhinal cortex varied as function of the dorso-ventral position of these cells, and that these densities paralleled their grid size. This finding suggests that activity regulates ion channel distribution profiles. Finally, ion channel distribution profiles can also be altered in a disease state (reviewed in Remy et al., 2010; Shah et al., 2010).

The described method can, in principle, be applied to any fluorescence signal together with a signal for neuronal morphology. We focused in this study on the analysis of the profiles of certain voltage-gated ion channels. Any ion channel subunit, even different splice variants, phosphorylated/non-phosphorylated, or membrane associated/non-associated forms could be labeled, provided that specific antibodies exist for them. Similarly to voltage-gated ion channels, the distribution of ligand-gated ion channels, or intracellular signaling molecules such as kinases could also be analyzed. For example, the voltage-range of activation for distal dendritic A-type K^+^ channels in CA1 apical dendrites is shifted to 10–15 mV hyperpolarized compared to that from the proximal dendrite/soma. This shift has been linked to a gradient in protein kinases along the apical dendrite, as kinase activity shifts the voltage-range of activation of the dendritic channels in the depolarized direction (Magee, 2008). The immediate early gene Arc (also known as Arg3.1), an important effector molecule for normal brain function and downstream of many signaling pathways, is probably synthesized locally at the sites of synaptic activity in the dendrites. Determining the distribution pattern of the Arc protein may shed light on which synapses have been activated or may undergo plasticity (reviewed in Shepherd and Bear, 2011). In addition, RNA distribution within neurons can be examined using fluorescent *in situ* hybridization. Hundreds of RNAs that have been found to be localized in dendrites, including structural proteins (MAP2), enzymes (aCaMKII), growth factors (BDNF and NT3), growth factor receptors (TrkA and TrkB), ligand-gated ion channels (glutamate and GABA receptor subunits), voltage-gated ion channels (calcium channels), and transcription factors (CREB) (Barrett and Eberwine, 2008). Finally, signals stemming from dyes that change their fluorescence properties as function of intracellular calcium or membrane voltage could also be analyzed in this way.

### Conflict of interest statement

The authors declare that the research was conducted in the absence of any commercial or financial relationships that could be construed as a potential conflict of interest.

## Appendix

**Table A1:** **Processing times and operator effort**.

**Procedure**	**Time**	**Operator effort**
Acquisition of confocal stack (scanning only)	5–30 min	30 s
Pre-processing: deconvolution	10–60 min	1–5 min
Pre-processing: conversion to 8-bit multilayer tif-file	2–5 min	2–5 min
Image processing: anisofilter	15–45 min	10 s
Image processing: segmentation	10–30 s	10 s
Inspection in ImageJ	1–2 min	1–2 min
DendriteTracker	5–10 min	1 min
Visual inspection in neuron	2–10 min	2–10 min
DendriteAnalyser	5–20 min	1–10 min

### Methods

#### Preparation of CVS-G pseudotyped SAD ΔG-eGFP and SAD ΔG-mCherry

CVS-G pseudotyped SAD ΔG vectors were prepared essentially as described in Rancz et al. (2011) with the exception that BSRT7/5 cells (Buchholz et al., 1999) were used instead of BHK-21 cells and pCAG-GS-CVS-G was used as the transcomplementing plasmid. BSRT7/5 cells, pCAG-GS-CVS-G, and viral starter stocks were a kind gift from K.-K. Conzelmann (Max-von-Pettenkofer Institute). Virus titers (~1 × 10^6^/ml) were determined by serial dilution of virus stocks and overnight infection of HEK-293T cells (essentially as described in Wickersham et al., 2010).

#### Antibodies

The following monoclonal antibodies against ion channel subunits were obtained from the UC Davis/NIH NeuroMab Facility: anti-Kv2.1 (K89/34), anti-Cav1.3 (N38/8), anti-cav1.2 (L57/46). Anti Kv2.1 has been validated using tissue from transgenic mouse lines in which the relevant channel subunit has been deleted. Additional antibodies were as follows: anti-GFP (rabbit polyclonal reference: A-11122, Life Technologies), anti-DsRed (rabbit polyclonal, reference: 632496, Clontech). Secondary antibodies (goat anti-rabbit and goat anti-mouse) conjugated to Alexa 488, 594, and 555 were purchased from Life Technologies.

#### Immunocytochemistry for Kv2.1

Cultured neurons were washed three times with ice cold 1 × PBS then fixed for 30 min on ice with 2% Paraformaldehyde/1 × PBS (essentially as described in Lim et al., 2000). Cells were washed three times with tris buffere saline (TBS), permeabilized for 5 min with 0.2% Triton X-100 in TBS, blocked in 3% bovine serum albumin (BSA), 4% normal goat serum (NGS) in 1 × TBS. Cells were incubated with primary antibodies [anti-Kv2.1 (1:200) and either anti-GFP (1:1000) or anti-Dsred (1:1000)] diluted in the same blocking buffer at 4°C overnight. Cells were rinsed three times with TBS, then incubated with secondary antibodies diluted 1:500 in 2% NGS, 3% BSA in TBS for 1 hour at RT. Cells were washed four times with TBS and then mounted in Prolong Gold Antifade reagent.

#### Immunocytochemistry for Cav1.2 and Cav1.3

Neurons were washed as above then fixed in 4% paraformaldehyde, 4% sucrose in 1 × PBS (as described in Zhang et al., 2006). Cells were then permeabilized with 0.2% saponin (Sigma). Immunological detection was performed as described for Kv2.1 with the exception that 1 × PBS was substituted for 1 × TBS. Anti-Cav1.2 and anti Cav1.3 were used at a dilution of 1:200.
